# The Pulse

**Published:** 1896-12-19

**Authors:** George Oliver


### THE PULSE.

By George Oliver, M.D., F.R.C.P. (Abstract of a Post-Graduate Lecture delivered at the Central London Sick Asylum.)

Long before the introduction of any instrumental aids, a great deal had been learned in regard to the pulse. Not only was it described in terms of speed and rhythm, but by assiduous study various qualities had been discriminated and practical deductions had been drawn both as to their meaning and their clinical importance. Thus the pulse was spoken of as hard or soft in regard to the resistance it offered to compression, as quick or slow in its duration, and as large or small in reference to the sensation of size conveyed to the finger; and many other nice distinctions had been made which need not here be mentioned. Of these qualities of the pulse, those of hardness and of softness were generally recognised as of primary clinical importance, the hard pulse being always regarded as pathological, and being often looked upon as an indication for venesection, the soft pulse showing the need for stimulants. The correctness of such distinctions, and even the truth of some of their empirical teachings, have in many cases been confirmed by modern instrumental research.

The graphic method has done much. It has shown reasons for facts which were previously known, and for shades of difference which were already empirically recognised, and has thus been of great educational and scientific utility; but it has proved of comparatively small clinical value as against the well-trained finger. The main facts which it demonstrated and made clear were in large degree already recognised by the *tactus eruditus* of the clinical observer.

A large range of new facts have, however, been recently opened up by the aid of the new instrumental methods which determine the calibre of the artery and the pulse-pressure, and these are of some importance from a clinical point of view.

As the result of many observations, it has been found, as, indeed, a moment's consideration would show must be the case, that the vascular system is capable of accommodating itself to the varying position of the body in such a manner as to counteract effects arising from the action of gravitation on the blood. Without some such special provision the vessels in the lower portions of the body would in the erect postures fill at the expense of those above, and every alteration of posture would involve an altered distribution of blood-supply. It is clear enough that in sitting or standing the hydrostatic pressure of the blood upon the walls of the vessels—its tendency, that is, to distend the vessels—is considerably greater in the lower than in the upper parts of the body, while in recumbency it is practically the same all over, and that if the blood vessels were inert tubes the radial artery would be smaller in the erect than in the recumbent posture.

In health, however, exactly the opposite is the case, the necessity of keeping the brain supplied with blood setting up such an action of the vaso-motor nervous system as to counteract this tendency, and, by squeezing blood out of other areas, to fill the vessels of the upper extremity to an even greater degree on sitting or standing than on lying down. This is a vital as opposed to a physical action; its absence is a definite departure from the normal, and by observing the extent to which patients suffering from a variety of disorders retain this power of accommodating the state of their vessels to varying posture, it has been possible to obtain from its presence or its absence many valuable hints as to the diagnosis and treatment of various obscure conditions.

By the calibre of an artery is meant the size of its lumen, and this is measured by the arteriometer, a mechanical arrangement for measuring the space traversed by a pad, while, squeezing the artery flat, *i.e.*, the distance from the point at which it comes in contact with the skin over the vessel to that at which the pulse is stopped. This distance indicates the diameter of the artery at the moment. Sensations conveyed by the finger are apt to be very illusory in this matter. It is difficult to analyse them, and in compressing an artery to distinguish between the pressure required to obliterate the pulse and the distance traversed by the finger in so doing. The arteriometer, however, is a measurer purely of size; for the estimation of pressure another instrument is used, which I have called the pulse pressure gauge (sphygmo-dynamometer), and by the use of these two instruments, which were designed to work with and to extend the sensory touch, we are enabled to ascertain with accuracy much that the finger fails to discern. These instruments are made by Mr. T. Hawksley, 357, Oxford Street.

Taking first the matter of the calibre of the artery, what is of interest is not so much its amount as its variation with altered posture. In the normal state the calibre enlarges when the patient assumes an erect posture (sitting or standing), the blood pressure being at the same time increased.

As a pathological condition, however, this variability in the calibre of the artery may (*a*) not occur, which we will call "fixation," or (*b*) may take place in the wrong direction, which we may conveniently term "reversal."

Fixation may be temporary or permanent, and, of course, may not be absolute, but may show itself only by a diminished range of variability. Temporary fixation occurs in many conditions, and is of very great clinical interest. It may be taken to mean an active vaso-motor constriction, and is found in migraine, in chronic gout, in passing gouty states in gouty people, and physiologically during the menstrual periods. The value of this sign is in some of these cases very considerable, for it may display the gouty origin of various complaints, the true nature of which might not otherwise be very clear. It is of interest also as in some sort explaining the frequent association of migraine with the menstrual period and with gouty states, the link between these various conditions lying in vaso-motor spasm.

Permanent fixation is not a matter of vaso-motor disturbance, but of organic change. It occurs in arterio-capillary sclerosis, and in all those organic changes which result in the production of increased arterial tension, such as renal disease and in atheroma, and thus may become an indication of a tendency towards senility, and it is met with in acquired syphilis.

Not much need be said regarding its occurrence in connection with renal disease, except that the very fact of its being found points strongly to the existence of sclerosis of the vessels rather than mere muscular hypertrophy as a cause of the accompanying high tension. In atheroma it is not surprising to find fixation of the vessels. It is, however, worth bearing in mind that atheroma occurs in two very different forms, which are differentiated in such investigations as I am now describing. In one the arteries are larger and in the other they are smaller than the normal, and considerable clinical experience leads to the conclusion that the "large" form is prognostically of much less serious import than the other.

The occurrence of fixation in acquired syphilis is of some importance. It has again and again led me either to prompt inquiry as to syphilis in the past history of a case, or to suggest that syphilis was or was not a probable factor in obscure cases. It has, for example, helped to clear up whether a chancre was in all probability infecting or not, and whether tabes or some other ailment of uncertain ætiology was or was not associated with syphilis. This curious fixity of the vessels, which obtains so commonly in syphilis, is probably due to an endarteritis set up at an early stage of the disease, which has induced contraction of the arteries—a prominent feature in acquired syphilis, especially after the lapse of years. It is worth bearing in mind that, so far as my observations go, while there is fixity of the vessels in albuminuria from renal disease, variability persists in functional albuminuria and in that from lardaceous kidney.

In "reversal" the maximum calibre occurs when in the recumbent posture. This is always an indication of depressed vitality in some form or another. It is exactly what would occur were the vascular system a dead set of tubes acted on by gravity alone, and its presence is an indication of a lessening of the activity of those vital processes by which the vascular system is controlled. Reversal, then, which might equally well be termed vaso-motor incompetence or inadequacy, occurs in all asthenic cases, in anæmia, in dilatation of the heart, and as a marked and essential feature of the case in neurasthenia.

Although it occurs in anæmia this condition is by no means necessary to its production. I have proved experimentally that this reversal of the normal relations takes place as a mere passing condition after a dose of nitro-glycerine or other vaso-dilator, in which case the fulness of the radials in this erect posture can be restored by pressing on the abdomen, and emptying the splanchnic area. It has been found that this procedure likewise rectifies the normal calibration in other cases of reversal. This maldistribution of the blood explains many of the most prominent symptoms met with in cases of neurasthenia—the head symptoms when in the erect posture and the gastric troubles with which such cases are constantly mixed up. "How well I can work when I lie down, and how soon I fail when I get up," says many a neurasthenic patient. The fact is that from vaso-motor incompetence the blood flows into his feet and his splanchnic area as soon as he gets up, and then his brain is starved. The indications as to treatment are sufficiently obvious, and the success which follows their adoption is often very great.

Time will not permit me to say much upon the second division of my subject, namely, the measurement of arterial tension. We are all accustomed by the application of the finger to ascertain the hardness or softness of the pulse. Probably by care and practice much more could be done in this way than is usually the case, but I feel convinced that there are great advantages in the use of instruments of precision for this purpose. A record of the actual pressure required to occlude the artery may be compared with other observations at a later date, and thus definite information can be obtained as to the progress of a case such as can be got in no other way. Too often, also, it is the case that observation by the finger takes note only of the maximum tension—that which occurs during what is spoken of as the tidal wave—to the neglect of that during the intervals between ventricular contractions. Now the meaning and importance of arterial tension depends almost as much on the relation between the maximum and the minimum as upon the exact pressure indicated by either, and while these points are indicated with accuracy by the pulse pressure gauge which I employ, the finger only conveys certain more or less vague sensations of "hardness" or "splashiness," as the case may be.

We all recognise the danger of that form of high tension which arises from growing peripheral resistance in renal disease and its accompanying cardiac hypertrophy, and it is not infrequently possible to forebode the occurrence of cerebral hæmorrhage in such cases. It is, however, not so generally borne in mind how serious may be small and often unrecognised degrees of tension when persistent, and when the cardiac power happens to be diminished by intercurrent illness. A heart which has been overcoming such a resistance so easily as not to have shown any appreciable signs of hypertrophy may fall just short of the burden thrown upon it when it has been weakened by an attack of influenza, diphtheria, or even tonsilitis; and in such cases any attempt to hasten convalescence may lead to dropsy and other symptoms of heart failure, the real cause of which may readily be mistaken, unless it be understood that even before the illness the heart was working to the limit of its powers. I need not, however, go further; the necessity of recognising high tension is now one of the commonplaces of medicine. The question is, How to do it? and for this purpose I recommend the use of such an instrument as the one I show you, partly because of the assistance it undoubtedly gives in arriving at an accurate conclusion, partly because of the great refinement in the finger sense which is obtained by constantly checking its conclusions by the record of the instrument, and partly because of the value which attaches to actual records in place of the fugitive impressions of the finger.

